# Machine learning landscapes and predictions for patient outcomes

**DOI:** 10.1098/rsos.170175

**Published:** 2017-07-26

**Authors:** Ritankar Das, David J. Wales

**Affiliations:** 1 Dascena, Foothill Blvd, Hayward, CA 94541, USA; 2 University Chemical Laboratories, Lensfield Road, Cambridge CB2 1EW, UK

**Keywords:** energy landscapes, machine learning, neural networks, patient mortality

## Abstract

The theory and computational tools developed to interpret and explore energy landscapes in molecular science are applied to the landscapes defined by local minima for neural networks. These machine learning landscapes correspond to fits of training data, where the inputs are vital signs and laboratory measurements for a database of patients, and the objective is to predict a clinical outcome. In this contribution, we test the predictions obtained by fitting to single measurements, and then to combinations of between 2 and 10 different patient medical data items. The effect of including measurements over different time intervals from the 48 h period in question is analysed, and the most recent values are found to be the most important. We also compare results obtained for neural networks as a function of the number of hidden nodes, and for different values of a regularization parameter. The predictions are compared with an alternative convex fitting function, and a strong correlation is observed. The dependence of these results on the patients randomly selected for training and testing decreases systematically with the size of the database available. The machine learning landscapes defined by neural network fits in this investigation have single-funnel character, which probably explains why it is relatively straightforward to obtain the global minimum solution, or a fit that behaves similarly to this optimal parameterization.

## 1. Introduction

There is an increasing need in the hospital setting for methods to predict the mortality risk and decompensation likelihood of patients [1]. Early identification of patient deterioration can assist provider organizations to properly manage and treat patients in a timely manner, to improve outcomes and allocate limited operational and financial resources efficiently [2,3]. With the rise in prevalence of electronic health records (EHRs) for clinical practice (over 75% of USA hospitals have a basic EHR system [4], while approximately 92% of UK hospitals use some form of EHR [5]) comes the opportunity to obtain clinically relevant patient data, such as vital signs and laboratory results, for patient instability forecasting-based decision support [6]. Additionally, with EHRs, patient health information is all centralized in one place, increasing ease of analysis with computational tools.

In this study, our aim is to predict the likelihood of mortality for critical care patients using approaches and analyses that build on the theory of potential energy landscapes in molecular science. Patient records were obtained from the Multiparameter Intelligent Monitoring in Intensive Care (MIMIC) III clinical database, which is a comprehensive dataset that contains clinical information of patients admitted to intensive care units [7]. Previous studies spanning epidemiology, clinical decision rules development, and electronic patient monitoring [8] have employed this database to investigate a broad range of areas in addition to mortality forecasting, including sepsis [9–11], acute pancreatitis [12] and extubation failure (the inability to sustain spontaneous breathing after removal of an artificial airway) [13]. Prior work on mortality prediction largely involves the use of clinical decision support systems (CDSSs), which are information systems created for the purpose of improving clinical decision-making [14]. While CDSSs have great potential to transform clinical care, there are some implementation and design issues for present systems [15]. Most current prediction models are based on a synthesis of patient-specific information and physiological parameters [16]. This formulation means that the models make the assumption that risk factors are independent of one another, which makes the patient mortality prediction less sensitive, and therefore less accurate. Among the most popular CDSSs are the modified early warning score (MEWS), the sequential organ failure assessment (SOFA) and the simplified acute physiology score (SAPS II), which have only moderate accuracy, and have not proved to be especially beneficial for clinical use [2,17].

Our approach in the present contribution is to apply the conceptual and computational tools of potential energy landscape theory to the landscape defined by neural network fits to patient data for prediction of mortality. For molecules and condensed matter, the underlying potential energy surface in a specific electronic state encodes its dynamics, thermodynamics and structure. The lowest energy structure is the global minimum of the potential energy surface. Location of this global minimum can often be accomplished efficiently using global optimization techniques. In this study, likely candidates for global minima of neural network fits are located using basin-hopping global optimization [18–20].

For the neural network fitting, the cost function that is minimized is derived from the difference between the predicted and actual patient outcomes. Generally in the case of non-convex cost functions, there will be multiple local minima [21], and we can employ all the potential energy landscape methodologies to investigate emergent properties of the resulting machine learning landscape. Here, we illustrate how some of these tools from the potential energy landscape framework can be applied to analyse machine learning landscapes defined by a cost function involved in fitting to training data. Our aim is to showcase how energy landscape approaches can be applied to time series of medical data, and offer new perspectives for future research that may lead to improved predictive methods.

## 2. Machine learning fits and predictions

**Figure 1.**
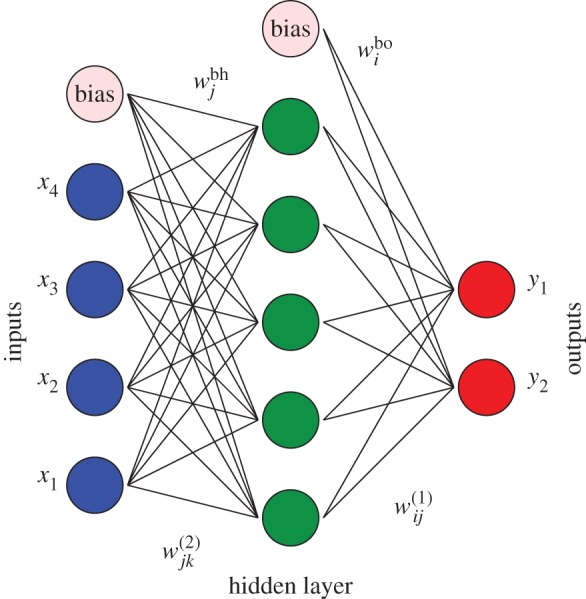
A three-layer neural network with four inputs, five hidden nodes and two outputs. The training variables are the link weights, $w_{jk}^{\left(2\right)}$ and $w_{ij}^{\left(1\right)}$, and the bias weights, *w*^*bh*^_*j*_ and *w*^bo^_*i*_.

The first fitting functions considered were three-layer neural networks (figure 1) containing input, output and hidden nodes [22]. We employed a single hidden layer in this initial survey in view of successful previous applications [23], and a bias was added to the sum of weights used in the activation function for each hidden node, $w_{j}^{\mathrm{bh}}$, and each output node, *w*^bo^_*i*_ [22]. The inputs correspond to patient data for a selected set of vital signs and laboratory measurements, including values from one or more of the 48 h time windows. We, therefore, consider a variable number of inputs, *N*_in_, for each patient, data point *α*, represented as ${\text{x}}^{\alpha }=\{x_{1}^{\alpha },\ldots ,x_{N_{\mathrm{in}}}^{\alpha }\}$, with the complete input dataset written as **X**={**x**^1^,…,**x**^*N*_data_^}.

There are *N*_out_=2 outputs, 0 and 1, corresponding to death in hospital and survival, which are calculated as

2.1 $$y_{i}^{\mathrm{NN}}=w_{i}^{\mathrm{bo}}+\sum_{j=1}^{N_{\mathrm{hidden}}}w_{ij}^{\left(1\right)}\tanh\left[w_{j}^{\mathrm{bh}}+\sum_{k=1}^{N_{\mathrm{in}}}w_{jk}^{\left(2\right)}x_{k}\right],$$

for each patient dataset **x** and network parameters $w_{ij}^{\left(1\right)}$ between hidden node *j* and output *i*, and $w_{jk}^{\left(2\right)}$ between input *k* and hidden node *j*, and bias weights *w*^bh^_*j*_ and *w*^bo^_*i*_.

To reduce the effect of outliers, the two outputs were transformed into softmax probabilities as

2.2 $$p_{c}^{\mathrm{NN}}(\text{W};\text{X})=\frac{e^{y_{c}^{\mathrm{NN}}}}{\left(e^{y_{0}^{\mathrm{NN}}}+e^{y_{1}^{\mathrm{NN}}}\right)}.$$

To train each network, we minimize the cost (objective) function, *E*^NN^(**W**;**X**), with respect to the variables $w_{ij}^{\left(1\right)}$, $w_{jk}^{\left(2\right)}$, $w_{j}^{\mathrm{bh}}$ and *w*^bo^_*i*_, written collectively as a vector of weights **W**. Basin-hopping global optimization was used [18–20] to search for the global minimum, and all the distinct minima obtained during these searches were saved for later comparison. In this approach, we take steps between local minima of the cost function, accepting or rejecting moves according to a simple Metropolis criterion [24] based upon the change in cost function, scaled by a parameter that plays the role of temperature. Downhill moves are always accepted, and the probability of accepting an uphill move depends on the fictitious temperature [18–20]. For the machine learning landscapes considered in the present work, locating the global minimum is straightforward, and the choice of basin-hopping parameters is not critical. A customized L-BFGS optimization routine was employed for local minimization, based on the limited memory version [25,26] of the quasi-Newton Broyden [27], Fletcher [28], Goldfarb [29], Shanno [30], BFGS procedure. Analytic first and second derivatives (including the regularization terms defined below) were programmed for both *E*^Q^(**W**;**X**) and *E*^NN^(**W**;**X**) in the public domain GMIN and OPTIM codes for exploration of the corresponding machine learning landscapes. Some further details are provided in §3, and a review of the energy landscape perspective in the context of machine learning has recently appeared [31].

For each patient, *α*, we know the actual outcome *c*(*α*)=0 or 1, and the cost function was written as

2.3 $$E^{\mathrm{NN}}(\text{W};\text{X})=-\frac{1}{N_{\mathrm{data}}}\sum_{\alpha =1}^{N_{\mathrm{data}}}\ln p_{c(\alpha )}^{\mathrm{NN}}(\text{W};\text{X})+\lambda {\text{W}}^{2},$$

which includes an *L*^2^ regularization term weighted by a specified coefficient *λ*>0. This term is designed to reduce overfitting, biasing against large values for individual variables. It also changes the zero eigenvalue, which would otherwise result from a uniform shift in all the *w*^bo^_*i*_, to 2*λ*.

The quality of predictions obtained from fits corresponding to local minima of *E*^NN^(**W**;**X**_train_) is judged from the corresponding values of *E*^NN^(**W**;**X**_test_), and especially from the area under the curve (AUC) values calculated from the probabilities $p_{c}^{\mathrm{NN}}(\text{W};{\text{X}}_{\mathrm{test}})$.

We have compared selected neural network results with those obtained from a quadratic function of the inputs, which is a convex function:

2.4 $$y_{i}^{Q}=w^{\left(0\right)}(i)+\sum_{k=1}^{N_{\mathrm{in}}}w_{k}^{\left(1\right)}(i)x_{k}+\sum_{k=1,j\geq k}^{N_{\mathrm{in}}}w_{kj}^{\left(2\right)}(i)x_{k}x_{j},$$

which has *N*_out_(1+*N*_in_(*N*_in_+3)/2) variables that we again write collectively as **W**. Probabilities were defined as

2.5 $$p_{c}^{Q}(\text{W};\text{X})=\frac{e^{y_{c}^{Q}}}{\left(e^{y_{0}^{Q}}+e^{y_{1}^{Q}}\right)},$$

with cost function

2.6 $$E^{Q}(\text{W};\text{X})=-\frac{1}{N_{\mathrm{data}}}\sum_{\alpha =1}^{N_{\mathrm{data}}}\ln p_{c(\alpha )}^{Q}(\text{W};\text{X})+\lambda {\text{W}}^{2}.$$

## 3. Machine learning and energy landscapes

In several recent contributions, we have shown how the energy landscape framework developed for molecular and condensed matter can be applied to cost functions that support multiple minima (non-convex functions) in machine learning [32,33]. Further discussion can be found in an overview article [31], which attempts to draw together various research strands that may be related using the ideas and computational tools of energy landscape theory [34].

Details of the methods employed to explore the machine learning landscape can be found in earlier work; these techniques are well established for applications to atomistic (and coarse-grained model) systems, and here we simply summarize the present calculations. To identify the global minimum for the neural network fits, we employed basin-hopping [18–20] as implemented in the GMIN program [35], with a modified L-BFGS minimization algorithm [25] (limited memory quasi-Newton Broyden [36], Fletcher [37], Goldfarb [38], Shanno [39] approach). The convergence condition for each minimization was a root mean square gradient below 10^−6^ reduced units, with tighter convergence of 10^−10^ for the distinct minima at the end of each run. This threshold is sufficiently tight to distinguish different local minima by simply comparing the cost function value. Finding the global minimum for these machine learning landscapes is generally straightforward, and so a large fixed step size and temperature parameter in the accept/reject step were employed to obtain a survey of local minima. Relatively short runs of 1000 basin-hopping steps were used in each case, guided by tests in previous work [32,33]. The present results surveyed a wide range of input data and alternative fits, requiring nearly 40 000 basin-hopping global optimization runs for the neural network fits, and over 10 000 local minimizations for the convex quadratic function. These tests correspond to different combinations of patient data items, different regularization parameters *λ*, different time windows for the patient data, and hidden layers with alternative numbers of hidden nodes.

Knowledge of local minima is sufficient to gauge the predictive power of different fitting functions, but it does not define a landscape. To achieve this more detailed level of description, we must understand how the local minima are connected by transition states (saddle points of index one [40]). This analysis was performed for selected test cases, one of which is reported in more detail below. The results are not only of fundamental interest for comparison with molecular landscapes but also provide insight into the location of different minima by local minimization, and the efficiency of global optimization. All the transition state searches used the doubly-nudged [41,42] elastic band [43,44] approach to identify candidates for accurate refinement with hybrid eigenvector-following [45,46]. We employed the OPTIM [47] program to characterize transition states and pathways, and the PATHSAMPLE [48] program to organize the overall exploration of each connected landscape.

The ability to visualize the landscape has played a key role in identifying molecular and condensed matter systems with the ability to self-organize, and distinguishing them from glass-formers [34]. Here, we employ disconnectivity graphs [49,50], where efficient relaxation to the global minimum is associated with single-funnel landscapes [34]. Most of the machine learning landscapes we have investigated so far seem to fall into this category [32,33], and a further example is presented in §5. Once again, the machine learning landscapes we have characterized in the present investigation appear to be funnelled, which explains why global optimization, or locating low-lying minima of the cost function, is relatively easy.

## 4. Input data

In total, 53 211 patient records were employed from the MIMIC III clinical database [51], which consists of anonymized data for patients at the Beth Israel Deaconess Medical Center (BIDMC) in Boston. The Institutional Review Boards of BIDMC and the Massachusetts Institute of Technology waived the requirement for individual patient consent, as our study does not impact clinical care and all data were deidentified.

Each record analysed from MIMIC III contains a collection of up to 33 different clinical measurements, including vital signs, for a 48 h period of hospitalization. For each patient, there is also a record of two possible outcomes, namely death in hospital and early discharge. In this study, we aimed to predict death in hospital, corresponding to 3879 patients. Of these records, 458 were removed, since the recorded outcomes were contradictory, with early discharge also reported. Vital sign and laboratory measurements without times were removed (1.6% and 1.2%, respectively). The number of patients with records of each type and the abbreviations employed are summarized in table 1.

**Table 1.** The 33 distinct types of vital sign and laboratory measurements contained in the MIMIC III database. In each case, the abbreviation is listed together with the number of patients, in increasing order.

data	description	no. patients
HCT	haematocrit	46
troponin	troponin	792
cholesterol	cholesterol	2721
ALP	alkaline phosphatase	7861
FiO_2_	fraction of inspired oxygen	11 343
PaO_2_	partial pressure of oxygen in blood	12 666
PaCO_2_	partial pressure of carbon dioxide in blood	12 674
albumin	albumin	12 787
ALT	alanine aminotransferase	14 959
bilirubin	bilirubin	17 019
lactate	lactate	17 437
ADBP	ambulatory diastolic blood pressure	17 975
ASBP	ambulatory systolic blood pressure	17 977
HCO_3_	bicarbonate	18 467
SpO_2_	peripheral capillary oxygen saturation	21 745
pH	pH	22 417
urine	urine output	23 147
SaO_2_	oxygen saturation of arterial blood	28 849
INR	international normalized ratio	31 266
NIDBP	non-invasive diastolic blood pressure	34 384
NISBP	non-invasive systolic blood pressure	34 432
Mg	magnesium	35 425
BUN	blood urea nitrogen	37 032
creatinine	creatinine	37 044
TEMP	temperature	37 082
GCS	Glasgow coma scale	37 270
RR	respiratory rate	37 283
glucose	glucose	39 133
Na	sodium	39 376
K	potassium	39 564
WBC	white blood cell count	41 445
platelets	platelets	41 971
HR	heart rate	43 740

To treat data obtained at irregular time intervals, the measurements were averaged over the 48 time windows corresponding to consecutive hours, ordered backwards in time from the most recent results (index 1). Empty entries were set to the earliest non-zero average available. For each data type, an average value was calculated over all patients for every time range considered, and the inputs were scaled to shift the mean to unity in each case. This ‘feature scaling’ puts the inputs into a standardized range and the local minimizations that are run in the subsequent fitting exhibit better convergence properties.

Training and testing were performed by randomly dividing the patients into two disjoint sets of equal size (with one more entry for testing when the total number of patients was odd). For each local minimum *α* of *E*^NN^(**W**_*α*_;**X**^train^) or *E*^Q^(**W**_*α*_;**X**^train^), the quality of the predictions was judged from the area under the receiver operating characteristic (ROC) curve obtained with the testing data as *E*^NN^(**W**_*α*_;**X**^test^) or *E*^Q^(**W**_*α*_;**X**^test^). The ROC curve is a plot of the true positive rate, *T*_*pr*_, against the false positive rate, *F*_*pr*_, as a function of the threshold probability, *P*, for making a certain classification. *P* is the threshold at which the output probability *p*^NN^_0_(**W**;**X**) or *p*^Q^_0_(**W**;**X**) is considered large enough to predict that outcome 0 (death in hospital) will occur. Hence,

4.1 $$\begin{array}{rl} T_{\mathrm{pr}}(\text{W};\text{X};P) & =\frac{\sum_{d=1}^{N_{\mathrm{data}}}\delta_{c(d),0}\Theta (p_{0}^{\mathrm{NN}}(\text{W};\text{X})-P)}{\sum_{d=1}^{N_{\mathrm{data}}}\delta_{c(d),0}}\\ \text{and}\;\;F_{\mathrm{pr}}(\text{W};\text{X};P) & =\frac{\sum_{d=1}^{N_{\mathrm{data}}}(1-\delta_{c(d),0})\Theta (p_{0}^{\mathrm{NN}}(\text{W};\text{X})-P)}{\sum_{d=1}^{N_{\mathrm{data}}}(1-\delta_{c(d),0})}, \end{array}$$

where *Θ* is the Heaviside step function, *δ* is the Kronecker delta and *c*(*d*) is the outcome (class label, 0 or 1) for patient *d*. The area under the curve is obtained by numerical integration of

4.2 $$\mathrm{AUC}(\text{W};\text{X})=\int_{0}^{1}T_{\mathrm{pr}}(\text{W};\text{X};P)\;dF_{\mathrm{pr}}(\text{W};\text{X};P).$$

The AUC value is interpreted as the probability that the prediction will discriminate correctly between two patients chosen at random from the sets with different outcomes. AUC values between 0.7 and 0.8, 0.8 and 0.9, and 0.9 and 1 are often described as fair, good and excellent, respectively.

## 5. Results

An extensive set of tests was first run for the 33 individual medical data items (referred to as measurements below) using neural network fits over various time ranges, with 3, 4, 5 and 6 hidden nodes and *λ*=10^−5^. In total, 13 299 global optimization runs were performed for these combinations. Table 2 summarizes the time interval corresponding to the maximum AUC achieved in testing for each of the hidden node values. This survey revealed that changing the number of hidden nodes did not have much effect. The results where the highest AUC values approaching 0.7 are achieved generally correspond to readings taken near the end of the 48 h period. The effect of varying the time interval was investigated systematically for combinations of input data where better AUC values are achieved, as discussed below. Hence, further details of the results for single data types are omitted for brevity. The main purpose of this initial survey was to guide the selection of input data for improved predictions.

**Table 2.** AUC values for test set predictions of patient outcome using one vital sign or laboratory data item and neural network fits for 3, 4, 5 and 6 hidden nodes. These are the results for the highest AUC values for testing, sorted for 6 hidden nodes. Fitting and testing were performed for data obtained in each hour, ranging from the last (index 1) to the first (index 48), for three consecutive hours 1–3, 2–4, …, 46–48, (again counting backwards in time, so that hour 1 is the last entry, i.e. the most recent), and for hours 1–2, 1–4, 1–6, 1–8, 1–10, 1–12 and 1–24. For the longest range 1–24, the network with 6 hidden nodes was not considered. In each case, the regularization parameter *λ* was fixed at 10^−5^. The maximum AUC values are italicized.

hidden nodes	3		4		5		6			
data items	time	AUC	time	AUC	time	AUC	time	AUC	*N*^train^_data_	*N*^test^_data_
troponin	34–36	0.485	11–13	0.512	34–36	0.503	34–36	0.489	396	396
HCT	1–8	0.500	1–8	0.500	1–8	0.500	1–8	0.500	23	23
GCS	1–24	0.590	1–24	0.582	1–24	0.574	1–12	0.516	18 635	18 635
pH	1–24	0.538	1–24	0.534	1–24	0.534	39–39	0.526	11 208	11 209
SaO_2_	39–39	0.532	39–39	0.532	39–39	0.532	39–39	0.532	14 424	14 425
Mg	10–12	0.544	1–24	0.545	10–12	0.544	10–12	0.544	17 712	17 713
ALT	18–20	0.566	48–48	0.565	47–47	0.562	16–18	0.551	7479	7480
ALP	20–22	0.586	23–25	0.561	23–25	0.556	48–48	0.551	3930	3931
cholesterol	1–8	0.554	1–24	0.555	1–10	0.555	1–10	0.553	1360	1361
SpO_2_	48–48	0.544	3–5	0.556	3–5	0.557	3–5	0.558	10 872	10 873
bilirubin	13–15	0.580	4–6	0.569	1–4	0.568	41–41	0.562	8509	8510
K	4–6	0.552	1–12	0.561	1–12	0.561	1–12	0.566	19 782	19 782
TEMP	1–3	0.570	1–3	0.568	1–3	0.569	1–3	0.570	18 541	18 541
NISBP	1–12	0.576	1–6	0.578	1–8	0.575	1–8	0.578	17 216	17 216
Na	1–3	0.582	1–3	0.582	1–3	0.582	1–3	0.582	19 688	19 688
WBC	1–24	0.606	1–24	0.641	1–24	0.642	1–4	0.584	20 722	20 723
FiO_2_	1–10	0.622	1–24	0.612	1–12	0.613	27–27	0.590	5671	5672
platelets	1–24	0.632	1–24	0.631	1–24	0.638	1–12	0.598	20 985	20 986
albumin	41–41	0.580	35–37	0.594	36–38	0.606	23–25	0.602	6393	6394
lactate	1–10	0.553	31–31	0.596	31–31	0.603	31–31	0.602	8718	8719
PaO_2_	46–46	0.605	46–46	0.604	46–46	0.604	46–46	0.604	6333	6333
NIDBP	1–2	0.605	1–2	0.606	1–2	0.605	1–2	0.605	17 192	17 192
HR	1–3	0.603	1–3	0.604	1–24	0.611	1–3	0.607	21 870	21 870
glucose	1–3	0.598	1–12	0.621	1–12	0.620	1–12	0.619	19 566	19 567
INR	1–3	0.619	2	0.620	2	0.619	2	0.619	15 633	15 633
PaCO_2_	2	0.627	2	0.627	2	0.627	2	0.627	6337	6337
creatinine	1	0.627	1–24	0.632	8–8	0.628	8–8	0.628	18 522	18 522
RR	42–44	0.630	44–44	0.629	40–42	0.629	40–42	0.629	18 641	18 642
ASBP	1–8	0.634	1–10	0.635	1–6	0.641	1–6	0.639	8988	8989
ADBP	3–5	0.646	3–5	0.635	3–5	0.640	3–5	0.646	8987	8988
urine	1–3	0.664	1–3	0.666	1–3	0.659	1–3	0.668	11 573	11 574
BUN	1–3	0.697	1–3	0.697	1–3	0.696	1–3	0.697	18 516	18 516
HCO_3_	2–4	*0.700*	2–4	*0.700*	2–4	*0.700*	2–4	*0.700*	9233	9234

Of the 33 different vital sign and laboratory measurements, 22 were considered in the next phase of calculations, where all 231 combinations of two distinct measurements were used as input data. This subset was selected based on the performance of the individual inputs and the number of data records available. Neural networks with 3, 5 and 7 hidden nodes were considered for time windows corresponding to the last hour (index 1) and the last two, three, six and twelve hours (ranges 1–2, 1–3, 1–6 and 1–12). A total of 3465 basin-hopping global optimization runs were therefore performed, all for *λ*=10^−5^. The maximum AUC values observed for any time window with each of the three hidden layers are collected in table 3 for the best pairs of input data, together with the number of patients in the training and test sets. The best AUC values increase from 0.7 to 0.8 when two measurements are considered in the fits, and again there is little dependence on the number of hidden nodes. There is no significant advantage in using memory of measurements taken before the final data collection in the last hour.

**Table 3.** AUC values for test set predictions of patient outcome using two vital sign or laboratory data items and neural network fits for 3, 5 and 7 hidden nodes. A total of 231 different combinations of three data items were considered; these are the results for the highest AUC values, sorted on the AUC values for 7 hidden nodes. Results were obtained for time ranges of 1 and 1–2, 1–3, 1–6 and 1–12 h using *λ*=10^−5^, and the highest AUC value is reported in each case, together with the corresponding time range. The maximum AUC values obtained are highlighted in italics for each choice of hidden nodes.

hidden nodes	3		5		7			
data items	time	AUC	time	AUC	time	AUC	*N*^train^_data_	*N*^test^_data_
urine INR	1–12	0.708	1–6	0.701	1–3	0.700	8348	8348
glucose BUN	1–12	0.704	1–6	0.699	1–6	0.700	18 365	18 365
WBC BUN	1	0.702	1	0.704	1	0.703	18 308	18 308
pH BUN	1	0.703	1	0.703	1	0.703	10 347	10 348
INR BUN	1–2	0.703	1	0.704	1	0.704	15 542	15 542
RR BUN	1–12	0.709	1–6	0.707	1–2	0.704	18 278	18 279
platelets BUN	1	0.707	1–2	0.708	1–2	0.705	18 362	18 363
urine HR	1–12	0.706	1–12	0.710	1–3	0.705	11 571	11 572
NISBP BUN	1–6	0.708	1–2	0.706	1	0.706	16 914	16 914
FiO_2_ BUN	1	0.711	1	0.713	1	0.707	4626	4626
bilirubin BUN	1	0.711	1	0.709	1	0.707	7245	7246
urine FiO_2_	1	0.668	1	0.682	1	0.711	1397	1398
NIDBP BUN	1–3	0.712	1–3	0.712	1	0.711	16 891	16 892
SpO_2_ BUN	1	0.712	1	0.713	1	0.712	10 622	10 623
HCO_3_ BUN	1–6	0.714	1–2	0.712	1–3	0.712	9229	9230
creatinine BUN	1–2	0.716	1–2	0.718	1–2	0.717	18 513	18 513
urine bilirubin	1	0.722	1	0.722	1	0.721	4658	4658
PaCO_2_ BUN	1–2	0.723	1–2	0.722	1	0.722	6205	6205
HR BUN	1–6	0.724	1–2	0.723	1–2	0.724	18 443	18 443
HCO_3_ ASBP	1–3	0.719	1–3	0.725	1–6	0.724	3791	3792
BUN ADBP	1–6	0.738	1–3	0.746	1–3	0.737	8885	8885
urine BUN	1–3	0.736	1–3	0.741	1–3	0.738	9830	9831
pH GCS	1	0.736	1	0.736	1–3	0.739	10 243	10 243
glucose GCS	1	0.737	1–6	0.743	1	0.741	18 367	18 368
BUN ASBP	1–6	0.745	1–6	0.748	1–6	0.745	8886	8886
NISBP GCS	1	0.751	1	0.748	1–2	0.747	17 167	17 167
GCS bilirubin	1–2	0.762	1–3	0.759	1–3	0.750	7088	7089
NIDBP GCS	1	0.751	1	0.752	1–2	0.752	17 143	17 144
HR GCS	1	0.761	1–2	0.759	1	0.760	18 582	18 582
platelets GCS	1–6	0.751	1–3	0.764	1	0.761	18 174	18 175
WBC GCS	1–3	0.754	1	0.764	1–3	0.763	18 096	18 096
SpO_2_ GCS	1–2	0.759	1–3	0.765	1–3	0.767	10833	10834
PaCO_2_ GCS	1	0.771	1	0.771	1	0.770	6227	6228
RR GCS	1	0.766	1–3	0.771	1–2	0.770	18 560	18 561
TEMP GCS	1–6	0.765	1–6	0.769	1–2	0.770	18 459	18 459
GCS ADBP	1–3	0.776	1	0.776	1	0.773	8941	8942
PaO_2_ GCS	1	0.775	1	0.778	1	0.777	6224	6225
GCS ASBP	1	0.777	1	0.781	1	0.780	8942	8943
GCS creatinine	1–3	0.773	1	0.783	1	0.780	18 244	18 244
INR GCS	1	0.783	1	0.781	1	0.784	15 512	15 512
GCS FiO_2_	1	0.779	1	0.782	1	0.787	4535	4536
urine GCS	1	0.793	1	0.795	1	0.793	9869	9870
HCO_3_ GCS	1	0.798	1–2	0.796	1–3	0.794	9166	9166
GCS BUN	1–3	*0.795*	1	*0.801*	1	*0.802*	18 238	18 239

All three possible combinations were then considered for the same 22 measurements as for the doublet combinations, with time intervals 1 and 1–2 and hidden layers of 3 and 5 nodes. These 1540 triplet combinations therefore required 6160 global optimization runs. In this case, the results were compared with the quadratic fitting function defined in equation (2.4), for time ranges 1–2, 1–3, 1–6 and 1–12, with *λ*=10^−5^. The results for the best combinations, sorted on the neural net values, and the time interval and number of hidden nodes they were achieved for, are collected in table 4. We see that using three data types raises the best AUC values to around 0.85, which is clearly a worthwhile improvement. The correlation between the best neural network and quadratic fit predictions is analysed further below.

**Table 4.** AUC values for test set predictions of patient outcome using three vital sign or laboratory data items. A total of 1540 different combinations of three data items were considered; these are the results for the highest AUC values, sorted on the neural network AUC values. For the quadratic fitting function, results were obtained for time ranges of 1 and 1–2, 1–3, 1–6 and 1–12 h. For the neural network fits, we considered time ranges of 1 and 1–2 with 3 and 5 hidden nodes (*N*_*h*_). The highest AUC values obtained for the test data amongst all the local minima obtained in training are reported in each case for *λ*=10^−5^ and the time range and number of hidden nodes they correspond to. The maximum AUC values obtained for any combination are highlighted in italics for each fitting function.

	neural network fit			quadratic fit			
data items	time	*N*_*h*_	AUC	time	AUC	*N*^train^_data_	*N*^test^_data_
WBC urine GCS	1	3	0.800	1–3	0.789	9569	9570
platelets HCO_3_ GCS	1–2	5	0.800	1–6	0.749	9111	9112
INR GCS ADBP	1	5	0.801	1–2	0.723	8091	8092
urine GCS ADBP	1	5	0.801	1–2	0.806	5520	5521
GCS creatinine ASBP	1	5	0.802	1	0.705	8851	8851
INR GCS ASBP	1	5	0.802	1–2	0.712	8092	8093
PaO_2_ FiO_2_ creatinine	1–2	3	0.802	1	0.649	393	393
TEMP GCS BUN	1	5	0.803	1–2	0.715	18 121	18 121
pH GCS BUN	1–2	5	0.803	1–2	0.731	10 153	10 154
GCS BUN bilirubin	1–2	5	0.804	1–12	0.769	7075	7075
RR GCS BUN	1	5	0.806	1–2	0.723	18 218	18 219
urine GCS FiO_2_	1	3	0.806	1	0.808	348	348
urine platelets GCS	1	5	0.806	1	0.795	9645	9645
INR GCS BUN	1–2	5	0.807	1–2	0.725	15 448	15 448
PaO_2_ GCS BUN	1	3	0.807	1–2	0.805	6154	6154
NIDBP GCS BUN	1	5	0.807	1–3	0.722	16 856	16 856
urine INR GCS	1	3	0.807	1–3	0.807	8330	8331
GCS creatinine BUN	1	5	0.808	1	0.716	18 236	18 237
WBC GCS BUN	1	5	0.808	1	0.724	18 052	18 053
PaCO_2_ GCS ADBP	1	3	0.809	1	0.804	5001	5002
platelets GCS BUN	1	5	0.809	1	0.718	18 104	18 105
urine GCS ASBP	1	3	0.810	1–3	0.811	5520	5520
PaCO_2_ GCS BUN	1	5	0.811	1	0.808	6157	6158
SpO_2_ GCS BUN	1	5	0.811	1–3	0.800	10 598	10 598
NISBP GCS BUN	1	5	0.814	1	0.720	16 878	16 879
GCS FiO_2_ ASBP	1	3	0.814	1	0.804	2934	2935
urine HCO_3_ GCS	1–2	3	0.815	1	0.803	943	943
GCS BUN ADBP	1	5	0.817	1–3	0.745	8847	8848
HR GCS BUN	1	5	0.818	1–2	0.733	18 238	18 239
HCO_3_ GCS BUN	1	5	0.819	1	0.786	9162	9163
SpO_2_ GCS FiO_2_	1	3	0.819	1	0.829	627	627
GCS FiO_2_ ADBP	1	3	0.821	1	0.791	2934	2934
urine GCS BUN	1–2	5	0.822	1–3	0.821	9672	9673
GCS BUN ASBP	1	5	0.822	1–3	0.759	8848	8849
GCS FiO_2_ BUN	1	3	0.823	1–12	0.820	4481	4481
PaCO_2_ GCS FiO_2_	1–2	3	0.827	1	0.839	413	413
HCO_3_ GCS ASBP	1–2	3	0.830	1–3	0.785	3771	3771
HCO_3_ GCS ADBP	1	3	0.839	1	0.791	3770	3770
PaO_2_ GCS FiO_2_	1	3	*0.853*	1	*0.848*	413	413

The number of distinct measurements was further reduced to 14 for calculations involving all possible quartet combinations. For the 1001 quartets, we considered time ranges 1 and 1–2 h, with 3, 5 and 7 hidden nodes for the neural net fits, requiring 6006 global optimization runs. In this case, we compared the quadratic fits for the same time ranges, along with regularization parameters of *λ*=10^−5^ and 10^−6^ (table 5). The results show that the maximum AUC values do not improve significantly from the best triplet combinations in table 4, and that there is no significant difference for the two values of the regularization parameter.

**Table 5.** AUC values for test set predictions of patient outcome using four vital sign or laboratory data items. A total of 1001 different combinations of four data items were considered; these are the results for the highest AUC values, sorted on the neural network AUC values for *λ*=10^−6^. Results were obtained for two *λ* values, time ranges of 1 and 1–2, and 3, 5 and 7 hidden nodes (*N*_*h*_) for the neural network fits. The highest AUC values for the test data among all the local minima obtained in training are reported in each case, along with the time range and number of hidden nodes they correspond to. The maximum AUC values obtained for any combination are highlighted in italics for each fitting function and *λ* value.

	neural network fit						quadratic fit					
	*λ*=10^−5^			*λ*=10^−6^			*λ*=10^−5^		*λ*=10^−6^			
data items	time	*N*_*h*_	AUC	time	*N*_*h*_	AUC	time	AUC	time	AUC	*N*^train^_data_	*N*^test^_data_
RR INR HCO3 GCS	1	5	0.819	1–2	5	0.818	1–2	0.781	1–2	0.781	7704	7704
platelets HCO_3_ GCS ASBP	1	5	0.823	1	5	0.818	1–2	0.781	1–2	0.779	3764	3765
TEMP PaCO_2_ GCS BUN	1	3	0.819	1	3	0.818	1	0.811	1	0.812	6157	6157
urine platelets PaCO_2_ GCS	1	5	0.821	1	5	0.819	1	0.810	1	0.810	5870	5870
urine HCO_3_ GCS creatinine	1	3	0.822	1	3	0.820	1	0.836	1	0.838	943	943
HR GCS creatinine BUN	1	7	0.820	1	7	0.820	1	0.734	1	0.734	18 236	18 237
RR platelets GCS BUN	1	7	0.817	1	7	0.820	1–2	0.727	1–2	0.727	18 085	18 086
platelets INR GCS BUN	1	7	0.823	1	5	0.820	1	0.735	1	0.735	15 413	15 414
urine INR GCS BUN	1	5	0.819	1	7	0.820	1	0.812	1	0.812	8283	8283
RR GCS creatinine BUN	1	5	0.818	1	7	0.820	1–2	0.723	1–2	0.723	18 216	18 217
RR HCO_3_ GCS BUN	1–2	3	0.819	1	5	0.821	1–2	0.794	1–2	0.792	9158	9158
INR HCO_3_ GCS BUN	1	5	0.823	1	7	0.822	1	0.783	1	0.783	7706	7706
RR PaCO_2_ GCS BUN	1	3	0.821	1	5	0.822	1	0.819	1	0.819	6151	6152
platelets HR GCS BUN	1	7	0.820	1	5	0.822	1	0.737	1	0.737	18 104	18 105
pH GCS BUN ASBP	1	5	0.825	1–2	5	0.822	1	0.771	1	0.771	7738	7738
HR HCO_3_ GCS BUN	1	5	0.824	1	7	0.822	1	0.791	1	0.790	9162	9163
urine GCS creatinine ASBP	1	3	0.825	1	5	0.823	1	0.826	1	0.826	5446	5446
RR HR GCS BUN	1	7	0.821	1	7	0.823	1–2	0.743	1–2	0.741	18 218	18 219
INR HCO_3_ GCS ASBP	1	5	0.832	1	5	0.823	1	0.786	1	0.785	3445	3445
RR HCO_3_ GCS ASBP	1	3	0.828	1	3	0.823	1	0.780	1	0.779	3768	3768
platelets HCO_3_ GCS BUN	1	5	0.820	1	7	0.824	1	0.782	1	0.781	9108	9109
GCS creatinine BUN ASBP	1	5	0.825	1	5	0.825	1	0.757	1	0.757	8847	8848
pH urine HCO_3_ GCS	1	3	0.763	1	3	0.826	1	0.835	1	0.825	538	538
RR GCS BUN ASBP	1	5	0.828	1	5	0.826	1–2	0.761	1–2	0.760	8839	8839
TEMP platelets GCS BUN	1–2	5	0.823	1	5	0.826	1–2	0.725	1–2	0.727	17 987	17 987
HR GCS BUN ASBP	1	5	0.822	1	5	0.826	1	0.755	1	0.754	8848	8849
urine RR GCS BUN	1	5	0.828	1	3	0.827	1	0.826	1	0.827	9657	9658
HR HCO_3_ GCS ASBP	1	3	0.828	1	5	0.827	1	0.792	1	0.792	3771	3771
urine RR HCO_3_ GCS	1	3	0.821	1	3	0.828	1	0.849	1	0.845	943	943
urine TEMP GCS BUN	1–2	3	0.829	1	5	0.829	1	0.824	1	0.824	9671	9671
urine SpO_2_ GCS BUN	1	3	0.828	1	7	0.830	1	0.827	1	0.826	9671	9672
SpO_2_ GCS BUN ASBP	1	5	0.836	1	5	0.832	1	0.830	1	0.831	5783	5784
PaCO_2_ GCS BUN ASBP	1	5	0.834	1	3	0.832	1–2	0.832	1	0.831	4947	4947
urine GCS creatinine BUN	1	7	0.834	1	7	0.832	1	0.828	1	0.828	9671	9672
urine HR GCS BUN	1	5	0.829	1	5	0.833	1	0.827	1	0.827	9672	9673
platelets GCS BUN ASBP	1	5	0.834	1	5	0.833	1	0.765	1	0.765	8808	8808
urine PaCO_2_ GCS BUN	1	5	0.834	1	5	0.834	1	0.830	1	0.830	5882	5883
INR GCS BUN ASBP	1	5	0.834	1	7	0.834	1	0.779	1	0.778	8057	8058
urine platelets GCS BUN	1	5	0.836	1	5	0.834	1	0.823	1	0.823	9594	9595
TEMP GCS BUN ASBP	1–2	5	0.834	1	5	0.839	1	0.769	1	0.770	8733	8734
urine INR HCO_3_ GCS	1	3	0.808	1	3	0.840	1	*0.858*	1	*0.858*	816	816
urine GCS BUN ASBP	1	7	*0.844*	1	5	0.840	1	0.837	1	0.837	5444	5444
HCO_3_ GCS BUN ASBP	1	3	0.840	1	3	*0.842*	1–2	0.813	1	0.813	3770	3771

To further test whether extended combinations of clinical and laboratory measurements might lead to improved predictions, a variety of additional inputs were considered, including up to 10 different measurements. Here, we again compared the neural network and quadratic fits, this time for *λ*=10^−6^. Results for 41 of these combinations are collected in table 6. Hidden layers of 3, 5, 7, 9 and 11 nodes were used for the neural networks, with time ranges of 1 and 1–2. The quadratic fits were run for time ranges 1, 1–2, 1–3, …, 1–12. The combination of extra measurements does not improve predictions significantly over the best quartets, which can be combined with most of the other data types to produce quintets that produce comparable predictions. The extra computational expense involved in adding more inputs, and fitting more parameters for longer time intervals and more hidden nodes, does not result in significant improvements.

**Table 6.** AUC values for test set predictions of patient outcome using 5 to 10 vital sign or laboratory data items. A total of 45 different combinations of data items were considered; these are the results for the highest AUC values, sorted on the neural network AUC values. For the quadratic fitting function, results were obtained for time ranges of 1 and 1–2, 1–3, 1–6, 1–7, 1–8, 1–9, 1–10, 1–11 and 1–12 h. For the neural network fits, we considered time ranges of 1 and 1–2 with 3, 5, 7, 9 and 11 hidden nodes (*N*_*h*_). The highest AUC values obtained for the test data among all the local minima obtained in training are reported in each case for *λ*=10^−6^ and the time range and number of hidden nodes they correspond to. The maximum AUC values obtained for any combination are highlighted in italics for each fitting function. In each case, $N_{\mathrm{data}}^{\mathrm{test}}=N_{\mathrm{data}}^{\mathrm{train}}$ or *N*^train^_data_+1; the highest AUC values obtained for each fitting function are highlighted in italics.

	neural network fit			quadratic fit		
data items	time	*N*_*h*_	AUC	time	AUC	*N*^train^_data_
urine platelets RR pH ASBP	1	7	0.655	1–4	0.633	4836
urine platelets RR pH SpO_2_	1	9	0.658	1–2	0.643	5938
urine HCO_3_ GCS ASBP HR	1	3	0.671	1	0.790	483
urine platelets RR pH HCO_3_	1–2	3	0.679	1	0.731	538
urine platelets RR pH INR	1–2	3	0.679	1–2	0.676	5362
urine platelets RR pH creatinine	1–2	3	0.680	1	0.673	5906
urine HCO_3_ RR GCS ASBP BUN TEMP PaCO_2_ SpO_2_	1	3	0.683	1	0.741	420
urine platelets RR pH PaCO_2_	1	5	0.697	1–2	0.687	5881
urine platelets RR pH HR	1–2	11	0.713	11	0.753	5938
urine platelets RR pH HCO_3_ GCS ASBP BUN INR	1	5	0.714	1	0.789	391
urine platelets RR pH TEMP	1–2	9	0.714	1–12	0.745	5938
urine platelets RR pH HCO_3_ GCS ASBP BUN INR creatinine	1	3	0.721	1–3	0.659	391
urine HCO_3_ GCS ASBP pH	1	3	0.723	1	0.760	422
urine platelets RR pH BUN	1–2	3	0.726	1–2	0.719	5906
urine HCO_3_ GCS ASBP RR	1–2	11	0.731	1–4	0.760	81
urine HCO_3_ GCS ASBP creatinine	1	3	0.733	1	0.766	483
urine platelets RR pH HCO_3_ GCS ASBP	1–2	3	0.744	1	0.764	421
urine HCO_3_ GCS ASBP INR	1	3	0.754	1	0.743	439
urine platelets RR pH HCO_3_ GCS ASBP BUN	1–2	3	0.755	1	0.781	421
urine platelets RR pH HCO_3_ GCS	1	3	0.756	1	0.782	536
urine HCO_3_ RR GCS ASBP BUN TEMP PaCO_2_	1	5	0.757	1	0.764	420
urine HCO_3_ RR GCS ASBP BUN TEMP PaCO_2_ SpO_2_ creatinine	1	3	0.757	1	0.777	420
urine HCO_3_ GCS ASBP SpO_2_	1	3	0.762	1	0.767	483
urine HCO_3_ GCS ASBP TEMP	1	3	0.763	1	0.780	483
urine GCS BUN ASBP HCO_3_	1	3	0.764	1	0.832	483
urine HCO_3_ RR GCS ASBP	1–2	3	0.765	1	0.803	483
urine HCO_3_ RR GCS ASBP BUN TEMP	1	3	0.771	1	0.751	483
urine HCO_3_ GCS ASBP platelets	1	3	0.779	1	0.827	482
urine HCO_3_ RR GCS ASBP BUN	1–2	11	0.788	1	0.861	483
urine platelets RR pH GCS	1	7	0.794	1	0.785	5921
urine HCO_3_ GCS ASBP PaCO_2_	1	3	0.820	1	0.805	420
urine HCO_3_ GCS ASBP BUN	1	3	0.828	1	0.810	483
urine GCS BUN ASBP platelets	1–2	5	0.828	1	0.826	5410
urine GCS BUN ASBP PaCO_2_	1	3	0.831	1	0.837	4796
urine GCS BUN ASBP SpO_2_	1–2	3	0.834	1	0.835	5443
urine GCS BUN ASBP HR	1	5	0.836	1	0.833	5444
urine GCS BUN ASBP creatinine	1	5	0.840	1	0.842	5443
urine GCS BUN ASBP TEMP	1–2	3	0.841	1–2	0.843	5444
urine GCS BUN ASBP INR	1	3	0.842	1	0.839	4942
urine GCS BUN ASBP pH	1	9	0.851	1–6	0.842	4826
urine GCS BUN ASBP RR	1	9	*0.856*	1–7	*0.858*	5437

To provide some idea of the likely systematic error involved in these calculations, the runs were repeated for all the multiplet combinations reported in table 6 with 3 hidden nodes for the neural networks and measurements corresponding to the final hour (index 1). The patient data for training and testing were randomized differently for each repeat, and the mean and standard deviation of the AUC values obtained in testing were calculated for five (neural networks) or 10 (quadratic fits) independent choices. These results are collected in table 7. The standard deviation generally decreases for combinations with more patients, whether the predictions are good or not. For the best AUC values, the standard deviation is usually reassuringly small. Larger AUC values can sometimes be obtained for smaller datasets, but these are due to larger fluctuations for the smaller number of patients. In choosing the optimal combinations of patient data for predictions, we should be guided by results where the population that contributes to the measurements is as large as possible.

**Table 7.** Mean and standard deviation of the AUC values for test set predictions of patient outcome using 4 to 10 vital sign or laboratory data items. A total of 45 different combinations of data items were considered for the last time interval (index 1 above) and 3 hidden nodes for the neural network fits, with *λ*=10^−6^. The statistics were obtained for five and 10 random training and testing selections for the neural network and quadratic fits, respectively.

	neural network fit		quadratic fit		
data items	*μ*	*σ*	*μ*	*σ*	patients
urine HCO_3_ GCS ASBP RR	0.617	0.081	0.669	0.081	162
urine platelets RR pH HCO_3_ GCS ASBP BUN INR	0.672	0.070	0.722	0.049	783
urine platelets RR pH HCO_3_ GCS ASBP BUN INR creatinine	0.720	0.051	0.688	0.082	783
urine HCO_3_ GCS ASBP PaCO_2_	0.782	0.063	0.800	0.037	841
urine HCO_3_ RR GCS ASBP BUN TEMP PaCO_2_	0.708	0.042	0.786	0.045	841
urine HCO_3_ RR GCS ASBP BUN TEMP PaCO_2_ SpO_2_	0.711	0.037	0.744	0.033	841
urine HCO_3_ RR GCS ASBP BUN TEMP PaCO_2_ SpO_2_ creatinine	0.749	0.013	0.695	0.061	841
urine platelets RR pH HCO_3_ GCS ASBP	0.728	0.023	0.778	0.017	843
urine platelets RR pH HCO_3_ GCS ASBP BUN	0.725	0.045	0.804	0.038	843
urine HCO_3_ GCS ASBP pH	0.764	0.025	0.812	0.029	844
urine HCO_3_ GCS ASBP INR	0.754	0.031	0.782	0.038	878
urine HCO_3_ GCS ASBP platelets	0.770	0.021	0.816	0.044	964
urine GCS BUN ASBP HCO_3_	0.765	0.062	0.827	0.027	966
urine HCO_3_ GCS ASBP	0.759	0.049	0.826	0.038	966
urine HCO_3_ GCS ASBP BUN	0.755	0.073	0.829	0.029	966
urine HCO_3_ GCS ASBP creatinine	0.748	0.015	0.812	0.028	966
urine HCO_3_ GCS ASBP HR	0.746	0.052	0.806	0.028	966
urine HCO_3_ GCS ASBP SpO_2_	0.756	0.012	0.796	0.025	966
urine HCO_3_ GCS ASBP TEMP	0.776	0.039	0.810	0.020	966
urine HCO_3_ RR GCS ASBP	0.751	0.041	0.791	0.036	966
urine HCO_3_ RR GCS ASBP BUN	0.738	0.067	0.823	0.033	966
urine HCO_3_ RR GCS ASBP BUN TEMP	0.765	0.023	0.806	0.038	966
urine platelets RR pH HCO_3_ GCS	0.713	0.044	0.762	0.025	1073
urine platelets RR pH HCO_3_	0.613	0.072	0.688	0.042	1077
urine HCO_3_ RR GCS	0.782	0.025	0.821	0.020	1886
urine GCS BUN ASBP PaCO_2_	0.833	0.004	0.834	0.005	9592
urine GCS BUN ASBP pH	0.829	0.012	0.830	0.006	9653
urine platelets RR pH ASBP	0.648	0.014	0.624	0.009	9672
urine GCS BUN ASBP INR	0.842	0.004	0.847	0.010	9885
urine platelets RR pH INR	0.666	0.008	0.662	0.009	10 725
urine GCS BUN ASBP platelets	0.829	0.008	0.838	0.005	10 821
urine GCS BUN ASBP RR	0.842	0.010	0.845	0.008	10 875
urine GCS BUN ASBP creatinine	0.843	0.008	0.843	0.006	10 886
urine GCS BUN ASBP SpO_2_	0.835	0.002	0.839	0.006	10 887
urine GCS BUN ASBP	0.839	0.004	0.836	0.006	10 888
urine GCS BUN ASBP HR	0.834	0.003	0.842	0.007	10 888
urine GCS BUN ASBP TEMP	0.837	0.004	0.839	0.006	10 888
urine platelets RR pH PaCO_2_	0.689	0.006	0.681	0.012	11 762
urine platelets RR pH BUN	0.732	0.008	0.729	0.008	11 812
urine platelets RR pH creatinine	0.690	0.010	0.673	0.007	11 812
urine platelets RR pH GCS	0.798	0.010	0.790	0.006	11 842
urine platelets RR pH TEMP	0.657	0.018	0.649	0.018	11 876
urine platelets RR pH	0.665	0.012	0.639	0.010	11 877
urine platelets RR pH HR	0.669	0.013	0.658	0.006	11 877
urine platelets RR pH SpO_2_	0.655	0.005	0.638	0.013	11 877

**Figure 2.**
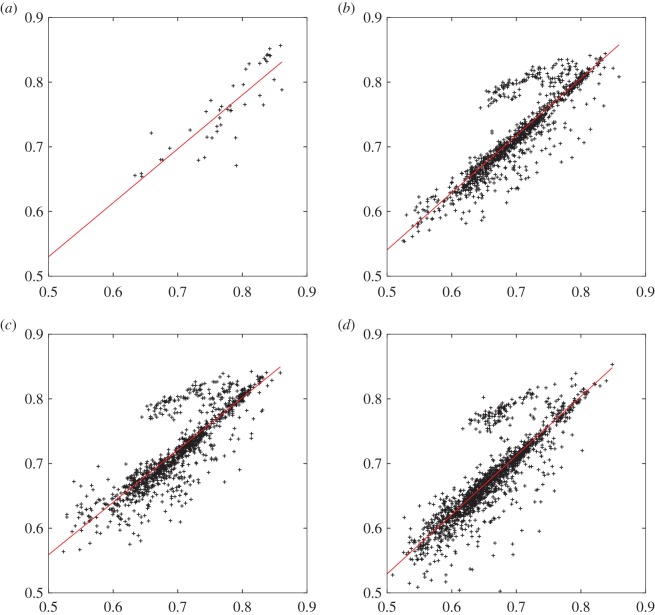
Plots of AUC values obtained in testing for neural networks (NNs) (vertical axis) against quadratic (Q) fits (horizontal axis) with different combinations of patient measurements. (*a*) Multiplets (five or more vital sign or laboratory data items) *λ*=10^−6^. (*b*) Four data items, *λ*=10^−5^. (*c*) Four data items, *λ*=10^−6^. (*d*) Three data items, *λ*=10^−5^. The maximum AUC testing value for any time interval and any number of hidden nodes (neural net fits) is used for each combination of measurements. The best fit straight line is plotted in each case.

Another check on the results is provided by the consistency of the predictions obtained from the neural network and quadratic fitting functions. To quantify this consistency the correlation coefficients for least-squares regression between the highest AUC values obtained in testing for the alternative fits were calculated for all four cases where common input data were considered. The resulting correlation coefficients are 0.86 for the multiplets (table 6 and figure 2*a*), 0.88 for the quartets with *λ*=10^−5^, (table 5 and figure 2*b*), 0.85 for quartets with *λ*=10^−6^, (table 5 and figure 2*c*) and 0.85 for the triplet combinations (table 4 and figure 2*d*). Here, the largest AUC value obtained in testing for any time window and number of hidden nodes (for the neural nets) was used. The quality of the predictions obtained from these alternative fitting functions exhibits quite a strong correlation. However, for the combinations of three and four different measurements, we note that there is a group of points where the best neural net prediction appears to be significantly better than the best quadratic fit result. This observation will be analysed in future work.

**Figure 3.**
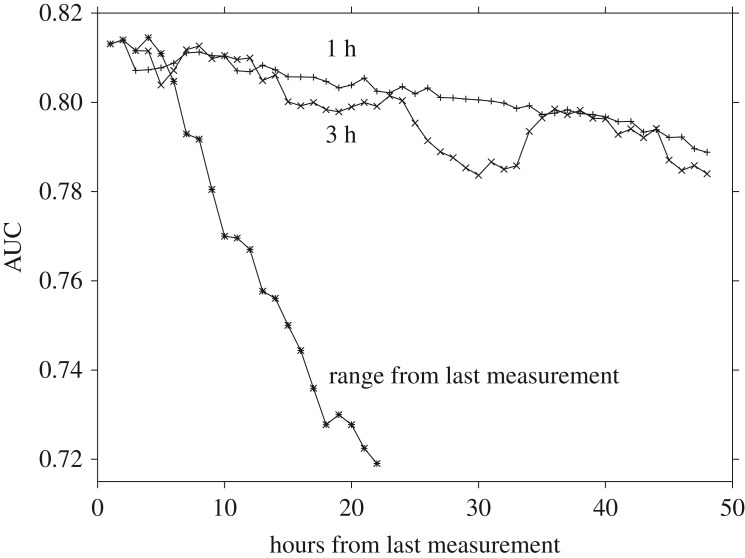
Plots of AUC values obtained in testing for the quadratic fitting function with four measurements (HCO_3_, GCS, BUN and ASBP) over different time windows. The three plots are for fits based upon each of the 48 h, blocks of three consecutive hours and a variable range from the last measurement including all hours backwards in time, i.e. 1, 1–2, 1–3, etc., up to 1–22. The training and testing sets included 3770 and 3771 patients, respectively, randomly reordered.

For one of the best combinations of input data, namely the HCO_3_, GCS, BUN, ASBP measurements, some further analysis of the landscape was conducted. To test the effect of using measurements from different time intervals, we employed the quadratic fitting function with *λ*=10^−5^. Training was performed for measurements corresponding to all 48 h, separately, the 46 possible blocks of three consecutive hours, and for all the measurements in the intervals 1–2, 1–3, 1–4, …, up to 1–22. The results are shown in figure 3. Here, we clearly see that it is the most recent measurements that produce the most accurate predictions in testing, and that there appears to be little or no advantage in considering earlier data.

**Figure 4.**
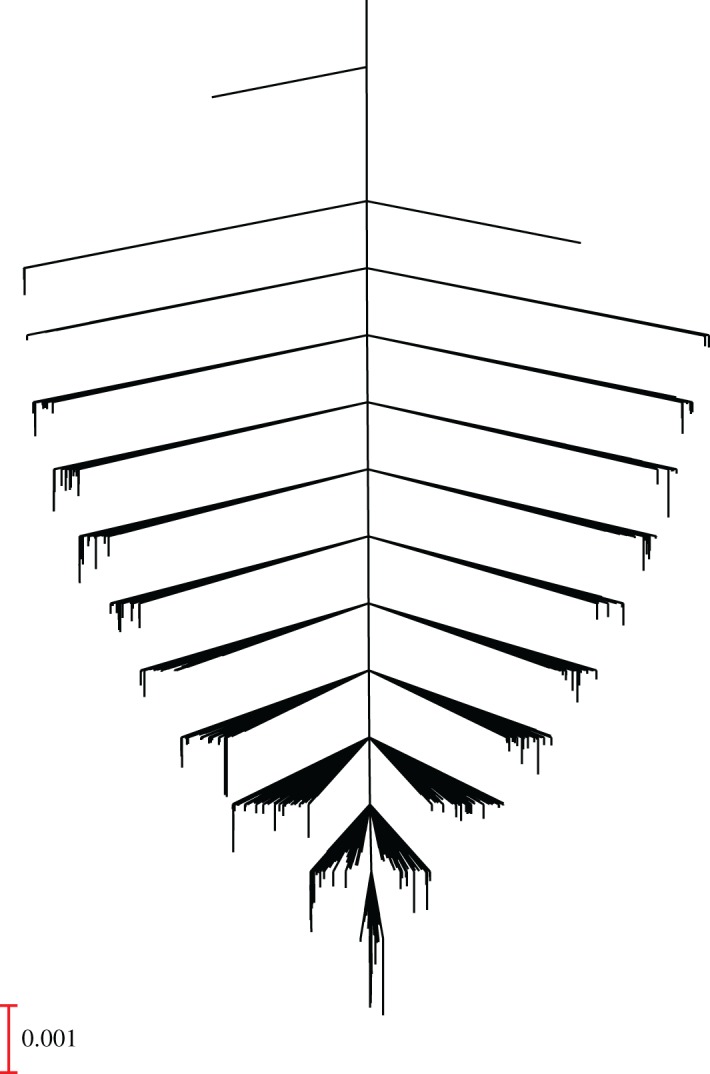
Disconnectivity graph for the machine learning landscape corresponding to the four measurements in figure 3 (HCO_3_, GCS, BUN and ASBP) for the final hour of data collection (index 1) with a neural network containing 5 hidden nodes, with *λ*=10^−5^. The stationary point database contains 1997 minima and 7492 transition states.

A disconnectivity graph for the HCO_3_, GCS, BUN, ASBP combination was constructed for a neural network containing 5 hidden nodes using the measurements from the final hour, with *λ*=10^−5^. The organization that results corresponds to a single-funnel landscape, as shown in figure 4. This structure means that local minima are connected to the global minimum by pathways with relatively low downhill barriers. There is no competing low-lying minimum separated from the global minimum by a high barrier, which would constitute a secondary funnel, corresponding to a kinetic trap for a molecular system. We identify this as an unfrustrated landscape [34,52–54], associated with efficient relaxation to the global minimum. Locating the global minimum using methods such as basin-hopping [18–20] is therefore straightforward. In a molecular system, this organization would also suggest that the low-lying minima are structurally similar. For the machine learning landscape, the funnelled character and lack of frustration suggests the absence of alternative fits with similarly high predictive quality, but a qualitatively different pattern of weights in the parameter space. This analysis is consistent with previous results involving machine learning classification of outcomes for geometry optimization in a simple molecular system [32]. A more detailed understanding of how the observed structure arises could be very insightful, and clearly merits further investigation. Such efforts are in progress, and will build upon analogues of thermodynamic and kinetic properties. For example, a theoretical framework to assign heat capacity features to local minima has recently been applied to both molecular and machine learning landscapes [55].

## 6. Conclusion

In this contribution, we have applied conceptual and computational tools from the potential energy landscapes framework to analyse machine learning landscapes defined by a cost function involved in fitting to training data. This approach has been used to predict patient outcomes using the MIMIC III clinical database [51]. We have compared results for neural networks with a range of different hidden nodes, as well as alternative regularization parameters. The predictive performance has been tested for various vital sign and laboratory measurements, including combinations of up to 10 different data types. We have also analysed how the predictions are affected when data from different time windows over the 48 h of collection are used for training and testing. Here, we find that the most recent measurements are generally the most useful, and including older data can degrade the results. Neural networks with between 3 and 11 hidden nodes were usually found to have similar performance, and the best AUC values obtained in testing are around 0.85 for particular combinations of three or more clinical measurements.

The neural network predictions have also been compared with an alternative quadratic fitting function, which exhibits a single minimum for a given set of training data. We find a strong correlation between the AUC values obtained from these different functions. To gauge the uncertainties in the patient outcome predictions, the calculations were repeated for both fitting functions using a number of different random choices of patients for the training and testing sets. The standard deviation in the resulting AUC values is lowest when the datasets are as large as possible.

Likely candidates for the global minima of the neural network fits are located using basin-hopping global optimization [18–20]. As in previous explorations of machine learning landscapes, short basin-hopping runs appear sufficient to reliably locate the lowest cost function value. To understand this observation, we have explored selected landscapes in more detail, characterizing the transition states that link local minima via steepest-descent paths, and the corresponding barriers defined by the cost function metric. These explorations produce the equivalent of a kinetic transition network [56–58] for a molecular or condensed matter system. The resulting databases of minima and transition states can be visualized using disconnectivity graphs [49,50]. One example is illustrated for a combination of four clinical measurements where a neural network with five hidden nodes exhibits good predictive properties. The underlying machine learning landscape has the appearance of a single funnel, which is the structure we associate with efficient relaxation to the global minimum, and self-organizing properties in atomistic systems [34,50,59]. Local minima are connected to the global minimum by relatively small downhill barriers. This organization probably explains why it is usually not difficult to find a fit corresponding to a low-lying minimum, with similar properties to the global minimum.

## Data accessibility

The software involved can be downloaded from URL www-wales.ch.cam.ac.uk. Input and output files will be deposited in the Cambridge Landscapes Database available at the same URL.
